# Objective Numerical Evaluation of Diffuse, Optically Reconstructed Images Using Structural Similarity Index

**DOI:** 10.3390/bios11120504

**Published:** 2021-12-08

**Authors:** Vicky Mudeng, Minseok Kim, Se-woon Choe

**Affiliations:** 1 Department of Medical IT Convergence Engineering, Kumoh National Institute of Technology, Gumi 39253, Korea; mudengvicky@kumoh.ac.kr; 2 Department of Electrical Engineering, Institut Teknologi Kalimantan, Balikpapan 76127, Indonesia; 3 Department of Mechanical System Engineering, Kumoh National Institute of Technology, Gumi 39177, Korea; 4 Department of Aeronautics, Mechanical and Electronic Convergence Engineering, Kumoh National Institute of Technology, Gumi 39177, Korea; 5 Department of IT Convergence Engineering, Kumoh National Institute of Technology, Gumi 39253, Korea

**Keywords:** diffuse optical tomography, structural similarity, human visible perception, numerical evaluation, biosensors

## Abstract

Diffuse optical tomography is emerging as a non-invasive optical modality used to evaluate tissue information by obtaining the optical properties’ distribution. Two procedures are performed to produce reconstructed absorption and reduced scattering images, which provide structural information that can be used to locate inclusions within tissues with the assistance of a known light intensity around the boundary. These methods are referred to as a forward problem and an inverse solution. Once the reconstructed image is obtained, a subjective measurement is used as the conventional way to assess the image. Hence, in this study, we developed an algorithm designed to numerically assess reconstructed images to identify inclusions using the structural similarity (SSIM) index. We compared four SSIM algorithms with 168 simulated reconstructed images involving the same inclusion position with different contrast ratios and inclusion sizes. A multiscale, improved SSIM containing a sharpness parameter (MS-ISSIM-S) was proposed to represent the potential evaluation compared with the human visible perception. The results indicated that the proposed MS-ISSIM-S is suitable for human visual perception by demonstrating a reduction of similarity score related to various contrasts with a similar size of inclusion; thus, this metric is promising for the objective numerical assessment of diffuse, optically reconstructed images.

## 1. Introduction

Diffuse optical tomography (DOT) is a promising imaging technology designed to reconstruct absorption and reduced scattering coefficients by obtaining the light propagation intensity around a tissue boundary [1]. DOT involves two major steps to accomplish the entire process of obtaining the optical property map distribution, including measurement and computation procedures. In the measurement system, several pairs of light sources and detectors are attached around a subject or phantom model to acquire the light radiance distribution. In the computation procedure, a reconstruction image algorithm is utilized to predict optical properties inside the tissue [2,3]. The DOT technique is an invasive modality because it uses the near-infrared (NIR) spectral window to image the structural and functional properties of human tissue. For this reason, several works with clinical and computational elements have explored three measurements, referred to as continuous-wave (CW), frequency-domain (FD), and time-domain (TD) [4,5,6]. A CW-DOT works with only the light intensity attenuation; thus, the direct current power voltage may be implemented to drive the associated laser source; meanwhile, an FD-DOT applies an amplitude-modulated light source with a typical frequency of 100 MHz. In contrast, to reduce the ill-posedness of CW-DOT and FD-DOT, TD-DOT, which produces photons with a time-of-flight distribution, can propagate in three regimes, such as ballistic, snake, and diffused photon patterns. It is considered possible to perform improved measurements via the TD-DOT method [7,8].

To complete the entire DOT computation, a forward problem based on the finite element method (FEM) and an inverse solution with a regularization algorithm must be solved [9]. Once these two procedures have been fulfilled, the optical property distribution indicated by the reconstructed images can be obtained. The reconstructed images provide structural or functional information related to tissue conditions. In the case of breast imaging, the distribution map of optical properties offers information associated with the presence of tumors. To assess the reconstructed images from DOT, the subjective knowledge and insight of medical image analysis experts are required, which tend to be costly and time consuming. The medical image analysis technique relies normally on the mean square error (MSE), peak signal-to-noise ratio (PSNR), and contrast-to-noise ratio (CNR); however, these assessments often present inconsistencies with the human visual system (HVS) [10,11,12]. A contrast-and-size detail (CSD) analysis was developed to deal with contrast ratio and size, and exhibited the capability to separate visible and invisible inclusions [13,14]. However, CSD is not appropriate for human perception and lacks a threshold value to distinguish the presence of inclusions. To overcome this issue, a structural similarity (SSIM) index was first introduced in 2004 to accord with the HVS by considering luminance, contrast, and structure calculation [12]. Since then, SSIM has become more popular in the field of image quality assessment (IQA), even in biomedical and clinical applications [15,16,17,18,19].

In optical modality, SSIM is capable of evaluating image enhancement, whereas in microwave imaging, SSIM offers image inspection of the breast [20,21]. In addition, radiological image assessments for computed tomography and magnetic resonance imaging have been conducted [22,23,24,25]. Moreover, to detect dopamine from the alternation of pH and histamine, as well as radiotherapy, SSIM can be used to objectively assess images with the assistance of a reference image [26,27]. The abovementioned researches concern SSIM in the medical field because several reports have demonstrated that SSIM is feasible for improving the sensitivity based on the purpose by implementing image processing insight [28]. The multiscale SSIM (MS-SSIM) may be more flexible than the mean SSIM (MSSIM) because it provides multiscale image assessment with downsampling by two images in each iteration [29,30]. In addition, SSIM has been repeatedly improved, with several derivative methods developed, such as gradient-based SSIM (GSSIM), three-component weighting region, four-component weighting region, complex wavelet, and an improved SSIM with a sharpness comparison (ISSIM-S) [31,32,33,34,35]. In these advanced implementations, SSIM was transformed to demonstrate reasonable performance in assessing images without a reference and can be used for image decomposition, identifying inter-patch and intra-patch similarities, and deblurring IQA [36,37,38,39]. SSIM is used widely to evaluate images, including medical images; therefore, this study presents four types of SSIM as a computer-based observer to assess DOT reconstructed images. The emphasis of this research was to evaluate simulated images to avoid uncertainty in a practical environment. MSSIM, MS-SSIM, mean ISSIM-S (MISSIM-S), and multiscale ISSIM-S (MS-ISSIM-S) were utilized to compare homogeneity and heterogeneity. To the best of our knowledge, comparisons of those four types of SSIM are described for the first time for evaluating DOT reconstructed images. Additionally, MS-ISSIM-S is a novel, proposed image quality metric in this research. A comparison of four SSIM algorithms was conducted with 168 simulated reconstructed images involving the same inclusion position, as well as different contrast ratios and inclusion sizes. The proposed MS-ISSIM-S measure is the ISSIM-S modified by performing a multiscale technique, as presented in Section 2.4. To evaluate the performance of these four SSIMs, the mean opinion score (MOS) calculated with Spearman’s rank correlation, as described in Section 2.5, was completed.

The remainder of this study is organized as follows. Section 2 describes the methodology and Section 3 describes the results and discussions. Section 4 presents some final concluding remarks.

## 2. Methodology

**Figure 1:**
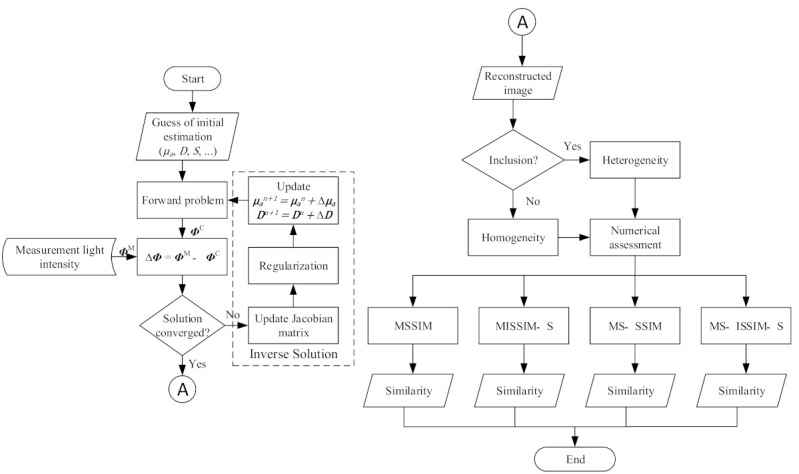
Block diagram of the process used to obtain similarity, where $\mu_{a}$ and D are absorption and diffusion coefficients, respectively, S denotes the light source, $\Phi^{M}$ expresses measured photon propagation, and $\Phi^{C}$ is computed light from solving DE.

The procedure begins with an image reconstruction process that yields reconstructed images of optical properties. The initial estimation of the optical properties, light source intensity, modulation frequency, and speed of light in the diffusion media must be completed first to simulate the forward problem. Then, a forward problem algorithm is employed to calculate the light distribution around the boundary in comparison with the light intensity from the measurement. If the solution converges, the simulation is stopped. However, if the solution does not converge, an inverse solution algorithm is used along with regularization to update the absorption and diffusion coefficients related to the reduced scattering coefficient. This image reconstruction will occur continuously until the stop criterion is satisfied. When the reconstructed images are obtained, two images are included, which separately contain information on homogeneity and heterogeneity, which are then compared with four numerical analysis assessment methods, including MSSIM, MISSIM-S, MS-SSIM, and MS-ISSIM-S. These four numerical analyses result is in similarity values. Figure 1 shows a diagram of the processes used to yield the similarity measures investigated in this study.

A forward model with FEM is first discussed in Section 2.1. Additionally, Section 2.2 and Section 2.3 review the inverse solution along with Tikhonov regularization (TR) and IQA involving the original SSIM, MS-SSIM, and ISSIM-S, respectively. Section 2.4 describes the proposed numerical image analysis measure of MS-ISSIM-S, and Section 2.5 describes the correlation method in comparison with MOS by Spearman’s rank correlation.

### 2.1. Forward Problem

This study describes the forward model of FD-DOT to express the light intensity distribution $\Phi \left(r,\omega \right)$ at position r and light modulation frequency ω with the known absorption coefficient $\mu_{a}$, diffusion coefficient D, light source term $S_{0}$, and speed of light in the media c by solving the diffusion equation (DE), as given below.

(1) $$\nabla .D\left(r\right)\nabla \Phi \left(r,\omega \right)-\left[\mu_{a}\left(r\right)-\frac{i\omega }{c}\right]\Phi \left(r,\omega \right)=-S_{0}\left(r,\omega \right),\;$$

where D is defined as

(2) $$D=\frac{1}{3\left[\mu_{s}\left(1-g\right)+\mu_{a}\right]}=\frac{1}{3\left(\mu_{s}’+\mu_{a}\right)}\;,\;$$

where $\mu_{s}$ is the scattering coefficient, g denotes the average cosine of the scattering angle, and $\mu_{s}’$ refers to a reduced scattering coefficient.

To simulate the light distribution with DE, as in Equation (1), an FEM is implemented with the exact optical property values of $\mu_{a}$ and $\mu_{s}’$, as well as the known $S_{0}\left(r,\omega \right)$ and the boundary condition. This study adopted the mixed boundary condition, as shown in Equation (3). The FEM can be simulated using two procedures. The boundary condition is substituted into a weak form, and the Galerkin method can be performed.

(3) $$-D\nabla \Phi \cdot \hat{n}=\alpha \Phi ,\;$$

where $\hat{n}$ refers to the unit vector and α denotes the incorporated reflection as the result of the refractive index difference at the boundary. Therefore, the discrete equation in matrix can be expressed as

(4) $$\left[\begin{array}{cc} A_{ij}^{bb}-\alpha A_{ij}^{bb} & A_{ij}^{bl}\\ A_{ij}^{lb} & A_{ij}^{ll} \end{array}\right]\left\{\begin{array}{c} \Phi_{j}^{b}\\ \Phi_{j}^{l} \end{array}\right\}=\left\{\begin{array}{c} S_{j}^{b}\\ S_{j}^{l} \end{array}\right\},\;$$

where A denotes the optical property matrix, b refers to the boundary node, l is the internal node, and i and j are matrix indexes. Thus, Equation (4) can calculate the forward model Φ in the simple matrix from optical property·radiance=source.

### 2.2. Inverse Solution

Because the goal of DOT is to reconstruct the optical properties inside the tissue with the provided light intensity information around the boundary, the distribution $\chi^{2}$ can be obtained by minimizing the misfit differences between the photon propagation rate being investigated around the geometry $\Phi^{M}$ and light intensity from solving the DE with the estimated optical properties $\Phi^{C}$, as expressed in Equation (5).

(5) $$\chi^{2}=\|∆\Phi {\|}_{2}^{2}=∥∆\Phi^{M}-\Phi^{C}{\|}_{2}^{2}.\;$$

These data-model misfit differences can be minimized by iteratively solving I∆χ=∆Φ, where $I=\left[\partial \Phi^{C}/\partial \mu_{a}\;\partial \Phi^{C}/\partial D\right]$ is the Jacobian matrix and ∆χ denotes $\left[∆\mu_{a};∆D\right]$, the optical coefficient of the update vector at each iteration. However, solving this inverse problem I∆χ=∆Φ usually involves the difficulty of an ill-posed problem as the number of model parameters increases. As a result, TR was introduced to overcome this issue. Hence, the inverse problem in DOT is formulated as an optimization of the damped least-squares problem.

(6) $$\underset{∆\chi }{\min}\left\{\|I∆\chi -∆\Phi {\|}_{2}^{2}+\lambda^{2}\|∆\chi {\|}_{2}^{2}\right\},\;$$

where λ is a regularization parameter. One can minimize this damped least-squares problem iteratively and then solve the following updated equation,

(7) $$\left(I^{T}I+\lambda^{2}I\right)∆\chi =I^{T}∆\Phi ,\;$$

where I is an identity matrix [40].

### 2.3. Image Quality Assessment

This section describes the three IQAs used herein. Section 2.3.1 reviews the original SSIM, Section 2.3.2 discusses MS-SSIM, and Section 2.3.2 describes ISSIM-S.

#### 2.3.1. Structural Similarity Index

SSIM was first introduced to overcome issues related to IQA. Previously, to measure the image quality, the MSE, SNR, PSNR, and CNR were commonly used. Nonetheless, these techniques are not suitable for human perception; in particular, MSE can generate the same value for two distorted images, even though one image is more visible than the other. In addition, SNR, PSNR, and CNR are attractive given their mathematical simplicity and clear physical meaning. In addition, CSD is promising for evaluating an image according to a comparison in terms of contrast and size but does not accord with the HVS because it shows occasional inconsistency related to contrast [10,12,14]. Therefore, SSIM emerged to overcome these issues, aiming to accord closely with human visual perception by considering the luminance l, contrast c, and structure s.

**Figure 2:**
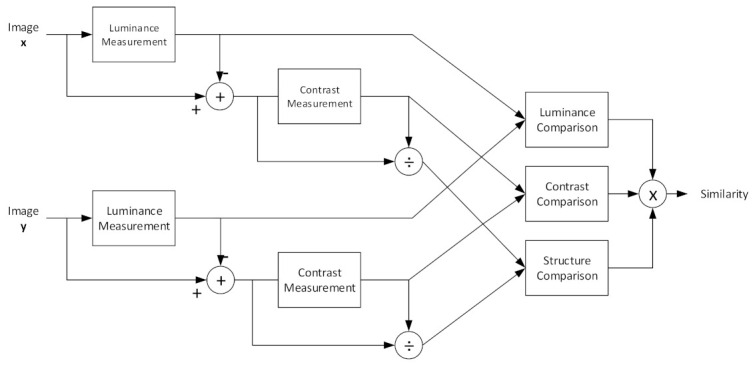
SSIM diagram to demonstrate the measurement procedure.

Figure 2 shows a diagram of the original SSIM. Luminance is calculated first over the two images. Image x is the homogeneous, reconstructed image as a reference, whereas the image y denotes a given reconstructed image under the test. The contrast is then measured. To obtain the structure, the covariance between x and y must be calculated. When these three parameters have been acquired, the combination produces a similarity score ranging from −1 to 1. However, in many cases, the similarity is between 0 and 1.

An SSIM score is a combination of comparison from the l, c, and s by calculating the mean intensity $\mu_{x}$ and $\mu_{y}$ along with standard deviation $\sigma_{x}$ and $\sigma_{y}$ for images x and y, as well as the covariance $\sigma_{xy}$ between x and y. The SSIM can be formulated as

(8) $$SSIM\left(x,y\right)={\left[l\left(x,y\right)\right]}^{\alpha }\cdot {\left[c\left(x,y\right)\right]}^{\beta }\cdot {\left[s\left(x,y\right)\right]}^{\gamma },\;$$

by setting α=β=γ=1 with

(9) $$l\left(x,y\right)=\frac{2\mu_{x}\mu_{y}+C_{1}}{\mu_{x}^{2}+\mu_{y}^{2}+C_{1}},\;$$

(10) $$c\left(x,y\right)=\frac{2\sigma_{x}\sigma_{y}+C_{2}}{\sigma_{x}^{2}+\sigma_{y}^{2}+C_{2}},\;$$

(11) $$s\left(x,y\right)=\frac{\sigma_{xy}+C_{3}}{\sigma_{x}\sigma_{y}+C_{3}},\;$$

where the constant $C_{1}={\left(K_{1}L\right)}^{2}$, $C_{2}={\left(K_{2}L\right)}^{2}$, $C_{3}=C_{2}/2$, and L=255. By setting $K_{1}$ and $K_{2}\ll 1$, the instability can be avoided at $\mu_{x}^{2}+\mu_{y}^{2}$ and $\sigma_{x}^{2}+\sigma_{y}^{2}$, and $\sigma_{x}\sigma_{y}$ are close to zero.

Nevertheless, SSIM performs well in the local statistics; hence, in this study, a 9×9 local window with the assistance of a Gaussian weighting function $w=\left\{w_{i}\;|\;i=1,\;2,\;3,\;\ldots ,\;N\right\}$, a standard deviation of 1.5, and the unit sum of $\overset{N}{\underset{i=1}{\sum }}w_{i}=1$ were used. Hence, the mean of SSIM can be expressed as

(12) $$MSSIM\left(X,Y\right)=\frac{1}{M}\overset{M}{\underset{j=1}{\sum }}SSIM\left(x_{j},y_{j}\right).\;$$

The window slides over the entire image, where X and Y are a reconstructed, homogeneous image and an examined reconstructed image, respectively, $x_{j}$ and $y_{j}$ are the image contents at the j-th local window, and M is the number of local windows in the image [12].

#### 2.3.2. Multiscale Structural Similarity

**Figure 3:**
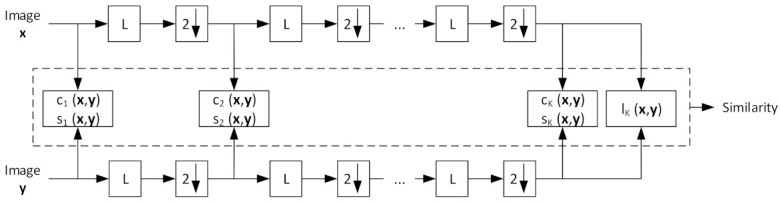
MS-SSIM diagram to demonstrate the measurement procedure. L denotes low-pass filtering and 2↓ refers to down-sampling by 2.

To improve the original SSIM, greater flexibility in viewing MS-SSIM was introduced, and the proposed measure showed outstanding performance compared to single-scale SSIM. Figure 3 shows a diagram of the MS-SSIM measurement. The measurement is simple, as a single-scale SSIM. First, images x and y are processed as in the original SSIM to yield the c and s in the first scale, and, in this case, the first scale is used as the original image size. Second, the low-pass filter (LPF) is deployed over the entire image and downsampled by 2. This assessment is repeated until K-scale, and the similarity is obtained by calculating the products of c and s in multiscale with the final l.

The entire MS-SSIM score can be evaluated using a combination of all the measurements via

(13) $$MS-SSIM\left(x,y\right)={\left[l_{K}\left(x,y\right)\right]}^{\alpha_{K}}\overset{K}{\underset{k=1}{\prod }}{\left[c_{k}\left(x,y\right)\right]}^{\beta_{k}}\cdot {\left[s_{k}\left(x,y\right)\right]}^{\gamma_{k}},\;$$

with $\beta_{1}=0.0448$, $\beta_{2}=0.2856$, $\beta_{3}=0.3001$, $\beta_{4}=0.2363$, and $\beta_{5}=0.1333$, where $\beta_{k}=\gamma_{k}=\alpha_{k}$ at k=1, 2, 3, …, K [30,34]. In this study, we set K=5.

#### 2.3.3. Improved Structural Similarity with Sharpness Comparison

The SSIM involves several shortcomings and, in such conditions, the similarity score is over-evaluated by comparing a reference image and images filtered by LPF. In contrast, slightly distorted images with geometrical transformations, such as spatial and rotation translations, have low similarity. Regarding these issues, SSIM may overestimate the filtered images. Hence, ISSIM-S was introduced to overcome these drawbacks [32]. In this study, the reconstructed images are similar to the blurred and translated images, as they were obtained by using different numbers of nodes and elements in the forward problem and an inverse solution to avoid the inverse crime.

**Figure 4:**
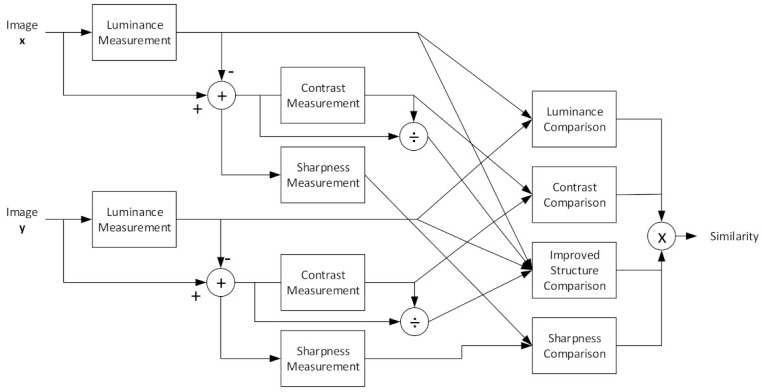
ISSIM-S diagram showing the measurement procedure.

Figure 4 shows a diagram of the ISSIM-S measurement. Compared with Figure 2, ISSIM-S has improvements in the sharpness and structure comparisons. The limitation of the SSIM is defined in Equation (11). The structure is sensitive to translation, rotation, and scaling; hence, a new structure comparison is necessary and can be formulated as

(14) $$\tilde{s}\left(x,y\right)=\frac{\left(2\sigma_{x-}\sigma_{y-}+C_{2}\right)\left(2\sigma_{x+}\sigma_{y+}+C_{2}\right)}{\left(\sigma_{x-}^{2}+\sigma_{y-}^{2}+C_{2}\right)\left(\sigma_{x+}^{2}+\sigma_{y+}^{2}+C_{2}\right)},\;$$

where $\sigma_{x-}$ and $\sigma_{y-}$ are the standard deviations for images x and y, which are smaller than $\mu_{x}$ and $\mu_{y}$, whereas $\sigma_{x+}$ and $\sigma_{y+}$ denote the standard deviations for images x and y, which are higher than $\mu_{x}$ and $\mu_{y}$. To decrease the overestimation, a new component, denoted as sharpness comparison $h\left(x,y\right),$ is utilized, which is correlated to the normalization digital Laplacian, as shown in Equation (15).

(15) $$h\left(x,y\right)=\frac{2\left|\nabla^{2}x\right|\left|\nabla^{2}y\right|+C_{2}}{{\left|\nabla^{2}x\right|}^{2}{\left|\nabla^{2}y\right|}^{2}+C_{2}},\;$$

where $\nabla^{2}x$ is the normalized digital Laplacian of image x, and $\nabla^{2}y$ is the normalized digital Laplacian of image y given by

(16) $$\nabla^{2}x=x-\mu_{x},\;$$

(17) $$\nabla^{2}y=y-\mu_{y}.\;$$

Then, the ISSIM-S and MISSIM-S are calculated as

(18) $$ISSIM-S\left(x,y\right)=l\left(x,y\right)\cdot c\left(x,y\right)\cdot \tilde{s}\left(x,y\right)\cdot h\left(x,y\right),\;$$

(19) $$MISSIM-S\left(X,Y\right)=\frac{1}{M}\overset{M}{\underset{j=1}{\sum }}ISSIM-S\left(x_{j},y_{j}\right).\;$$

### 2.4. Multiscale Improved Structural Similarity with Sharpness Comparison

The purpose of MISSIM-S is to decrease the overestimation of the blurred image and to approximate the similarity score with translation and scaling. Nevertheless, the reconstructed images are not only translation and scaling, but also aim to accord with the inclusion contrast and size. As noted above, MS-SSIM is effective because it can assess images at varying scales. By combining the principles of MISSIM-S and MS-SSIM, we propose MS-ISSIM-S as a new assessment technique in this work.

**Figure 5:**
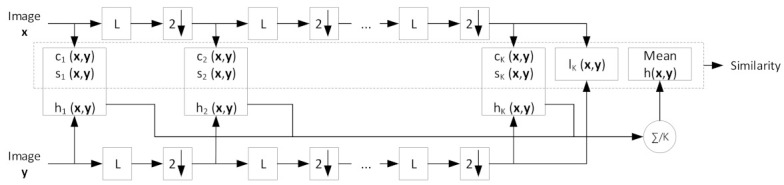
MS-ISSIM-S measurement procedure diagram. L denotes low-pass filtering and 2↓ refers to down-sampling by a factor of 2.

Figure 5 shows a diagram of the MS-ISSIM-S measurement. The entire procedure is similar to the assessment process in MS-SSIM, but h is calculated separately for each scale. Once the h is acquired at each scale, the mean of h is used to obtain the MS-ISSIM-S, as formulated in Equation (20).

(20) $$MS-ISSIM-S\left(x,y\right)=\left(\overset{K}{\underset{k=1}{\sum }}h_{k}\left(x,y\right)\right)\left({\left[l_{K}\left(x,y\right)\right]}^{\alpha_{K}}\overset{K}{\underset{k=1}{\prod }}{\left[c_{k}\left(x,y\right)\right]}^{\beta_{k}}\cdot {\left[s_{k}\left(x,y\right)\right]}^{\gamma_{k}}\right).\;$$

We adopted K=5.

### 2.5. Spearman’s Rank Correlation

To analyze the similarity score in each method, as explained in Section 2.3 and Section 2.4 with the HVS, a Spearman’s rank correlation [41] was used to determine the relationship between two independent variables. Correlation is a statistical method used to assess the association degree to aid in understanding the relationship between two variables, but not to distinguish the fundamental relation [42,43]. This correlation is a numerical value used to quantify the linear correlation between the MOS and reconstructed images analyzed for each SSIM type in this study. The correlation values are between −1 and 1, with −1 indicating a negative linear correlation, 0 expressing no relation, and +1 denoting a perfect linear correlation. This correlation measurement aims to identify the most appropriate method for the four SSIM methods used in this study. Spearman’s rank correlation coefficient can be obtained as

(21) $$\rho =1-\frac{6\sum_{p=1}^{P}d_{p}^{2}}{n\left(n^{2}-1\right)}\;,\;$$

where ρ denotes the correlation, $d_{p}$ expresses the difference in p-th rank between MOS and reconstructed images, and n is the number of reconstructed images in each case for every optical property, as stated in Section 3.1. In this case, n was 21. However, when there are ties in the rank, the correction factor cf is added as a summation to Equation (21); thus, it can be expressed as

(22) $$cf=\frac{m^{3}-m}{12}\;,\;$$

(23) $$\rho =1-\frac{6\left(\sum_{p=1}^{P}d_{p}^{2}+\frac{m_{1}^{3}-m_{1}}{12}+\frac{m_{2}^{3}-m_{2}}{12}+\ldots \right)}{n\left(n^{2}-1\right)}\;,\;$$

where $m_{1}$, $m_{2}$, … are element numbers in the tie ranks. These cf will be in numbers following the number of ties ranked.

## 3. Results and Discussions

To avoid uncertainty, we emphasized the use of reconstructed images from DOT simulations. Section 3.1 states the image reconstruction model by describing the results for the used simulation cases, and Section 3.2 defines the image assessment by showing the results for each used SSIM type, which were then compared with MOS by applying Spearman’s rank correlation.

### 3.1. Image Reconstruction Model

**Figure 6:**
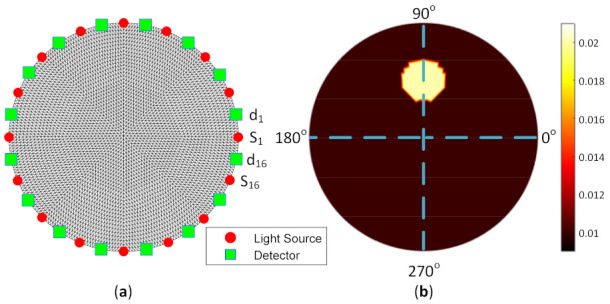
Model geometry showing (**a**) light sources (red dot) and detectors (green rectangle) where $S_{n}$ is the n -th sensor and $d_{n}$ denotes the n -th detector, as well as (**b**) artificial embedded inclusion position inside the tissue.

The simulated model used in this study was constructed to mimic breast tissue. A circular array with a group of finite element meshes comprised 4225 nodes, and 8192 triangle elements were implemented for the forward model. In addition, around the 80 mm of the diameter model boundary, 16 light sources and 16 detectors were attached to obtain the tissue information. Because there were 16 light sources and 16 detectors, the total measurement was 256 for the image reconstruction procedure. The total source detector (SD) was 32; hence, the distance between one source to another source and one detector to another detector was 22.5°. Moreover, the distance between SD was 11.25°. Figure 6a depicts the model geometry mesh designed to mimic breast tissue, with the attached red dots indicating light sources and the green rectangles as the detectors. $S_{1}$ indicates sensor 1, and $d_{1}$ denotes detector 1. The measurement began with $S_{1}$ as the light source, and then proceeded from $d_{1}$ to $d_{16}$ as the detectors to obtain the light intensity around the boundary. This measurement continued until $S_{16}$ as the light source penetrated the light inside the tissue model. Therefore, the measurement was rotated in a counterclockwise direction (CCW). Figure 6b shows the artificial embedded inclusion inside the tissue to mimic a breast tumor for exact $\mu_{a}$ distribution. The inclusion location and size were 90° and 7.5 mm in radius, respectively.

**Figure 7:**
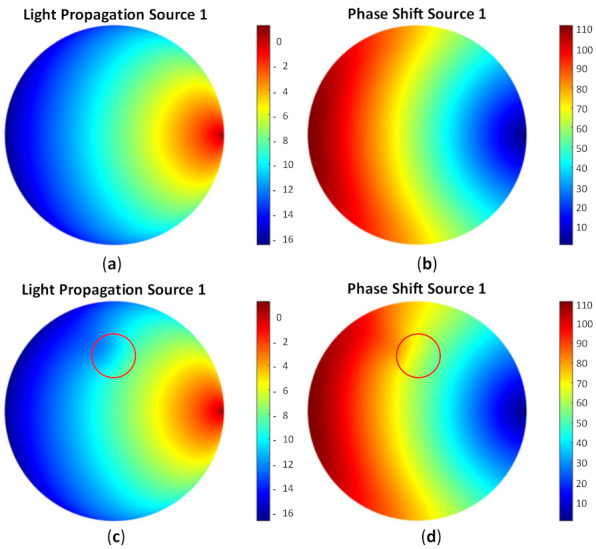
Distribution of (**a**) light propagation and (**b**) phase shift for homogeneous case; (**c**) light propagation and (**d**) phase shift for heterogeneous case, as in Figure 6b with the red circle, which shows the inclusion position when $S_{1}$ was used as the source.

To test the image reconstruction algorithm, a forward problem simulation was applied to acquire the light distribution. As the FD-DOT was implemented, it offered two solutions as the results, including light propagation and phase shift. Figure 7 depicts the light distribution map and the phase shift when $S_{1}$ was used as the source. Figure 7a,b shows the photon propagation and phase shift distribution for a homogeneity model. It may be observed that the light was distributed well inside the model by implementing the background $\mu_{a}=0.01{\;\mathrm{mm}}^{-1}$ and $\mu_{s}’=1{\;\mathrm{mm}}^{-1}$. Furthermore, Figure 7c,d depicts the light and phase shift distributions when the case shown in Figure 6b was applied. The red circles indicate inclusion locations. The distributions of the light source and phase shift inside the inclusion are recognized differently compared with the homogeneity case. These distributions verify that the inclusion exists in the specified position.

**Figure 8:**
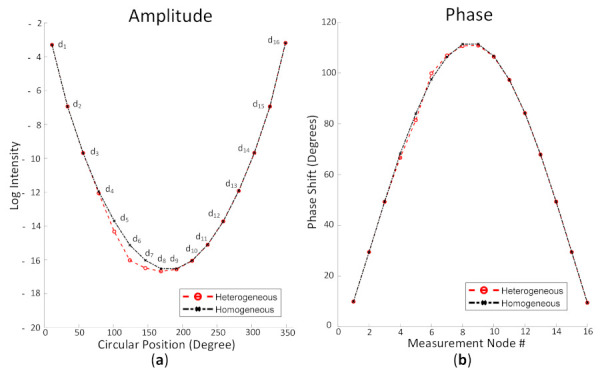
Obtained (**a**) light intensity and (**b**) phase shift from detectors attached around the model when $S_{1}$ was used as the source with a red, dashed line, indicating heterogeneity, and black, dashed line for homogeneity.

The log intensity measured around the boundary was inspected to confirm the results shown in Figure 7. Figure 8 depicts the light and phase shift around the boundary by using the simulated data from the detectors when $S_{1}$ was used as the source. Figure 8a demonstrates the intensity from $d_{1}$ to $d_{16}$. The light intensity from $d_{4}$ to $d_{9}$ indicated the differences in the intensity between the homogeneous (black, dashed lines) and heterogeneous (red, dashed lines) cases, whereas the phase shift was varied from $d_{4}$ to $d_{9}$, as shown in Figure 8b. These results indicated that the data around the boundary can be obtained by the DE method using the FEM. The next step was an inverse solution to yield the reconstructed optical property images.

To proceed with the entire process, an inverse solution was performed with different numbers of elements and nodes to avoid inverse crime; hence, 1536 triangular elements and 817 nodes were used. To achieve the purpose of this research, the reconstruction was performed in two cases representing homogeneous, invisible, and visible inclusions, as shown in Table 1. The representation of homogeneity in cases A1 and A2 was that the inclusion radius, $\mu_{a}$, and $\mu_{s}’$ were zero. To imitate invisible inclusion in case A1, the inclusion radius was 2.5 mm with $\mu_{a}=0.02\;{\mathrm{mm}}^{-1}$ and $\mu_{s}^{’}=2\;{\mathrm{mm}}^{-1}$, whereas the visible inclusion was 10 mm of the radius alongside $\mu_{a}=0.02\;{\mathrm{mm}}^{-1}$ and $\mu_{s}^{’}=3\;{\mathrm{mm}}^{-1}$. In contrast, for case A2, the unseeable inclusion used 2.5 mm of the radius along with $\mu_{a}=0.02\;{\mathrm{mm}}^{-1}$ and $\mu_{s}^{’}=0.89\;{\mathrm{mm}}^{-1}$, whereas the distinguishable inclusion used 10 mm as the radius, as well as $\mu_{a}=0.03\;{\mathrm{mm}}^{-1}$ and $\mu_{s}^{’}=0.89\;{\mathrm{mm}}^{-1}$.

**Figure 9:**
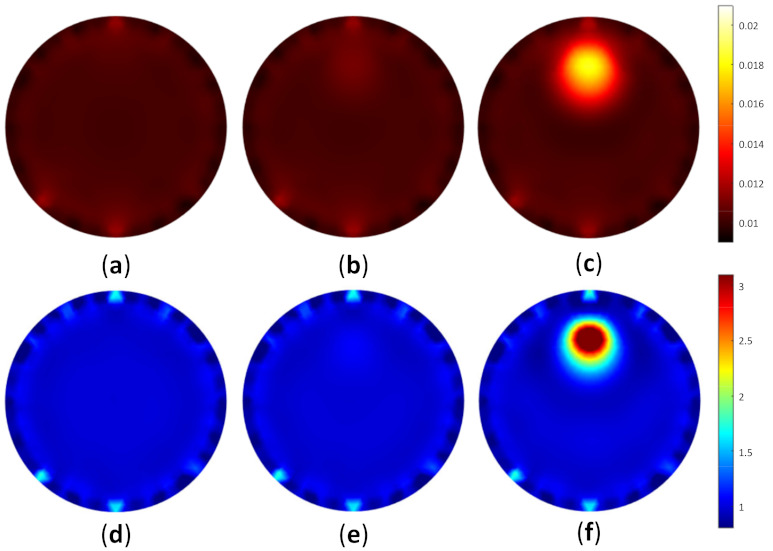
Reconstructed image of case A1, as shown in Table 1, representing (**a**) $\mu_{a}$ homogeneous, (**b**) $\mu_{a}$ invisible inclusion, (**c**) $\mu_{a}$ visible inclusion, (**d**) $\mu_{s}^{’}$ homogeneous, (**e**) $\mu_{s}^{’}$ invisible inclusion, and (**f**) $\mu_{s}^{’}$ visible inclusion.

**Figure 10:**
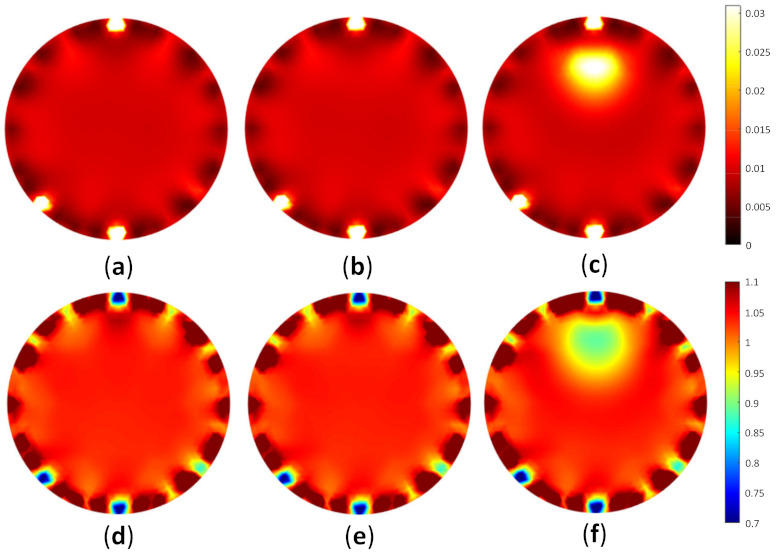
Reconstructed image of case A2, as shown in Table 1, representing (**a**) $\mu_{a}$ homogeneous, (**b**) $\mu_{a}$ invisible inclusion, (**c**) $\mu_{a}$ visible inclusion, (**d**) $\mu_{s}^{’}$ homogeneous, (**e**) $\mu_{s}^{’}$ invisible inclusion, and (**f**) $\mu_{s}^{’}$ visible inclusion.

**Figure 11:**
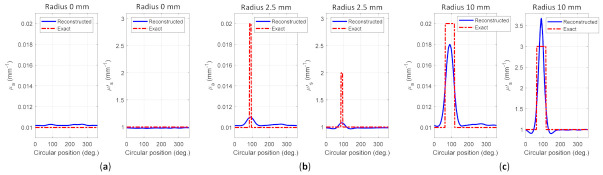
Circular profile of case A1 for (**a**) homogeneous, (**b**) invisible inclusion, and (**c**) visible inclusion.

**Figure 12:**
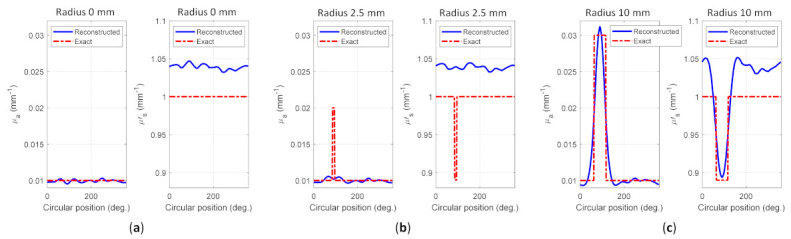
Circular profile of case A2 for (**a**) homogeneous, (**b**) invisible inclusion, and (**c**) visible inclusion.

Figure 9 depicts the reconstructed images for case A1, whereas Figure 10 shows the reconstructed images for case A2. Figure 9a–c and Figure 10a–c depict the reconstructed $\mu_{a}$ of homogeneous, invisible, and visible inclusions for cases A1 and A2, respectively, whereas Figure 9d–f and Figure 10d–f depict the reconstructed $\mu_{s}’$ of homogeneous, invisible, and visible inclusions for cases A1 and A2, respectively. These results indicated that the algorithm successfully reconstructed the optical properties; thus, it may be promising for the reconstruction using the other cases in simulation to accomplish the goal of this research. The circular profile used to examine the reconstructed images was applied over the image. Figure 11 and Figure 12 depict the circular profile for Figure 9 and Figure 10. Figure 11a a show the homogeneous circular profile for Figure 9a,d and Figure 10a,d. Moreover, Figure 11b and Figure 12b illustrate the invisible circular profile in Figure 9b,e and Figure 10b,e, whereas Figure 11c and Figure 12c show the visible circular profiles in Figure 9c,f and Figure 10c,f. These circular profiles demonstrated that 2.5 mm as an inclusion radius with a low-ratio contrast could not be reconstructed; however, with the large inclusion size with high contrast, it was possible to reconstruct the optical properties.

### 3.2. Image Assessment

As the results show in Section 3.1, the simulation proceeded to image assessment based on the computer observer using the reconstructed images to test numerical assessment with four types of SSIM: MSSIM, MS-SSIM, MISSIM-S, and MS-ISSIM-S. Hence, the objective decision numerically could be determined to distinguish between detectable and undetectable inclusions. Several uncertainties are encountered in medical image analysis; thus, medical image insight along with the medical background is required to prevent misconceptions, and individual or subjective assessments are highly preferable. This research attempted to manage the assessment method to analyze reconstructed images numerically; thus, the inspection could be more objective because the numerical method (computer-based observer) tended to be used. To avoid ambiguities in measuring the reconstructed DOT images, only simulated images were assigned in this study.

To obtain the reconstructed images, the cases shown in Table 2 were simulated with the inclusion location, as shown in Figure 6b. Additionally, 1% noise amplitude and 10% noise amplitude, phase, and optical properties were simultaneously completed to imitate the real environment. In this study, 168 reconstructed images involving 84 $\mu_{a}$ and $84\;\mu_{s}’$ images were used. The inclusion radii were 2.5, 3.75, 5, 6.25, 7.5, 8.75, and 10 mm with the same $\mu_{a}=0.02\;{\mathrm{mm}}^{-1}$ by changing $\mu_{s}^{’}=2$, 2.5, and $3\;{\mathrm{mm}}^{-1}$, namely, case B1. Case B2 had the same situation as case B1, but different optical properties, such as the same $\mu_{s}^{’}=0.89\;{\mathrm{mm}}^{-1}$ with $\mu_{a}=0.02$, 0.025, and $0.03\;{\mathrm{mm}}^{-1}$. A total of 672 assessments were performed.

**Figure 13:**
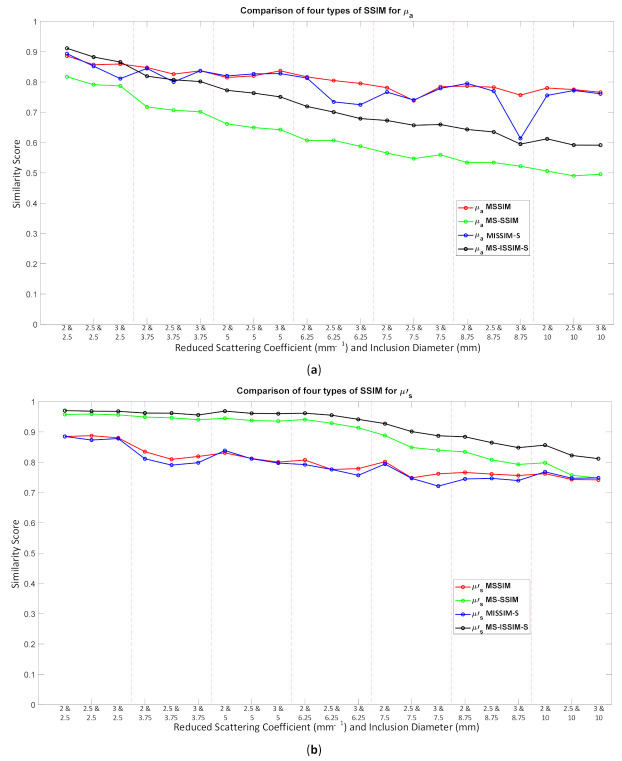
Four types of SSIM similarity score of case B1 with 1% noise amplitude for (**a**) $\mu_{a}$ and (**b**) $\mu_{s}’$.

**Figure 14:**
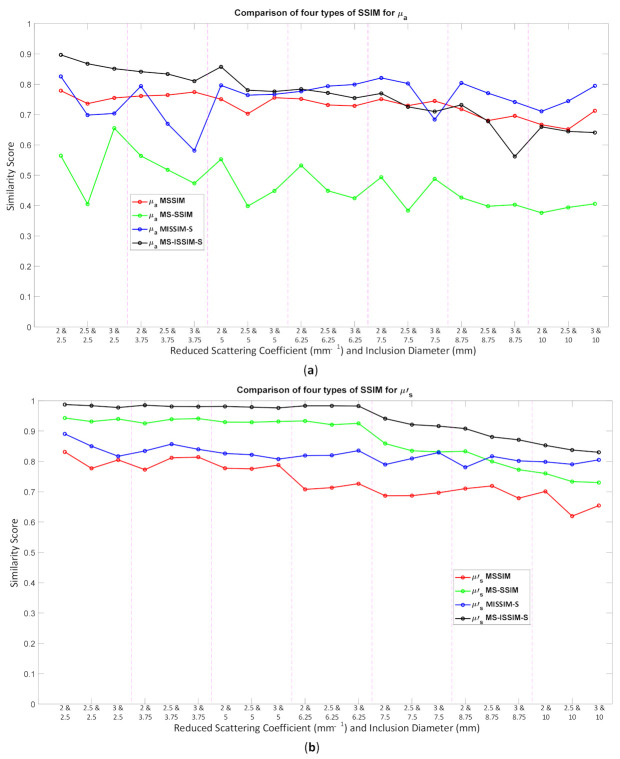
Four types of SSIM similarity score of case B1 with 10% noise amplitude, phase, and optical properties for (**a**) $\mu_{a}$ and (**b**) $\mu_{s}’$.

**Figure 15:**
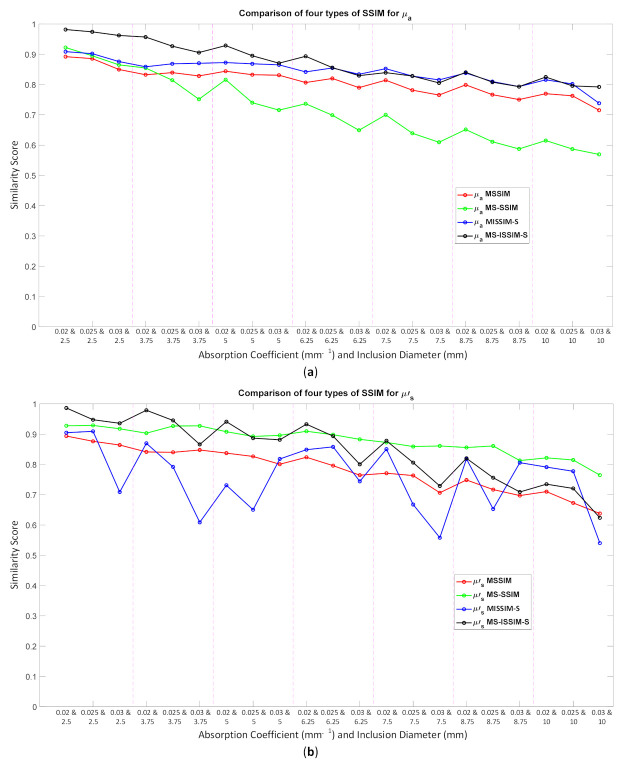
Four types of SSIM similarity score of case B2 with 1% noise amplitude for (**a**) $\mu_{a}$ and (**b**) $\mu_{s}’$.

**Figure 16:**
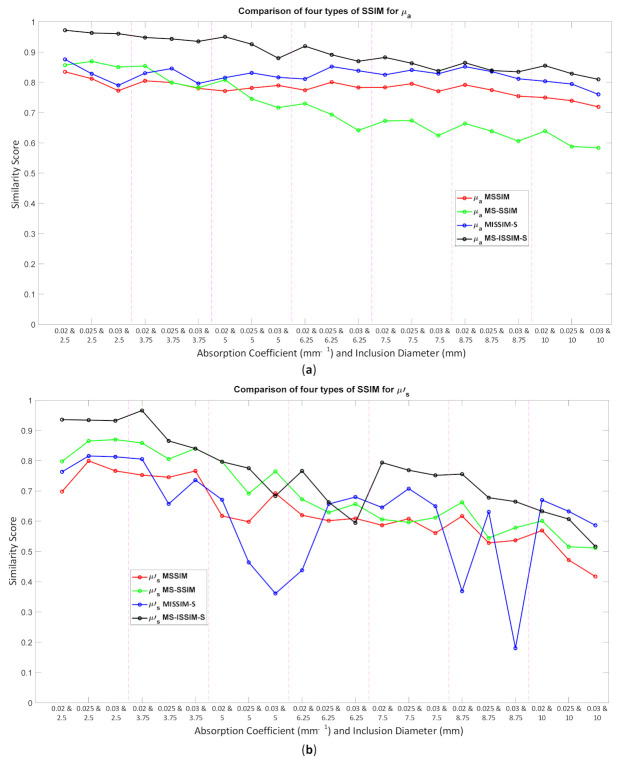
Four types of SSIM similarity score of case B2 with 10% noise amplitude, phase, and optical properties for (**a**) $\mu_{a}$ and (**b**) $\mu_{s}’$.

Because the SSIM is a full-reference image analysis, four reference images were necessary. Figure 9a,d was utilized as the $\mu_{a}$ and $\mu_{s}’$ reference images for case B1 with 1% noise amplitude and 10% noise amplitude, phase, and optical properties. Figure 10a,d was employed for case B2 with 1% noise amplitude and 10% noise amplitude, phase, and optical properties. Figure 13 depicts the comparison of the four types of SSIM evaluations for case B1 with 1% noise amplitude. The MS-ISSIM-S score decreased with the contrast ratio and inclusion size, as well as when every part, denoted by a magenta, dashed line, was examined in detail, and the similarity scores for MS-ISSIM-S showed a decreasing relationship with the contrast, as shown in Figure 13a. Meanwhile, for $\mu_{s}’$, MS-SSIM exhibited an almost similar trend to that of MS-ISSIM-S, as shown in Figure 13b. MSSIM and MISSIM-S demonstrated inconsistency in contrast and size. In addition, case B1 was observed with 10% noise amplitude, phase, and optical properties. Again, MS-ISSIM-S showed reliability related to the contrast and size for each part with the magenta, dashed line border, whereas MSSIM, MS-SSIM, and MISSIM-S were inappropriate with respect to contrast and size, and even similarities scores fluctuated, as shown in Figure 14a. MS-SSIM compared with MS-ISSIM-S, as shown in Figure 14b, had a slightly different trend, but showed inconsistency in contrast, whereas MSSIM and MISSIM-S were exceptionally inconsistent with the contrast and size. Figure 15a illustrates a similar tendency for MS-ISSIM-S and MS-SSIM when the contrast was increased. The similarity score must be decreased because the inclusion inside the tissue was to be detectable, as well as with the larger inclusion size. However, MSSIM and MISSIM-S were difficult to use in terms of contrast and size. Similar results are also shown in Figure 15b. To complete the entire computer-based observer evaluation, case B2 was simulated with 10% noise amplitude, phase, and optical properties. Even though the applied noise was 10%, the performance of MS-ISSIM-S was superior to that of MSSIM, MS-SSIM, and MISSIM-S because the similarity score was decreased with respect to the contrast and decreased relatively with the larger inclusion size, as shown in Figure 16a. Moreover, for $\mu_{s}’$, MS-ISSIM-S exhibited reasonable similarity scores associated with the contrast and size by showing that with the larger inclusion size and higher contrast the similarity was reduced. Nevertheless, MSSIM, MISSIM-S, and MS-SSIM were complicated to fit with the contrast ratio in each part, as shown in Figure 16b. As mentioned in Section 2.3.3, a MISSIM-S improved the MSSIM related to overestimation in the blurred images and underestimation with respect to translation and scaling; thus, it showed clearly, especially in Figure 16b, MSSIM overestimated the similarity score of reconstructed images, while MISSIM-S tried to suit inclusion size by presenting a lower similarity score when the $\mu_{s}^{’}=0.89\;{\mathrm{mm}}^{-1}$, with $\mu_{a}$ and inclusion sizes at $0.03\;{\mathrm{mm}}^{-1}$ and 5 mm, and $0.02\;{\mathrm{mm}}^{-1}$ and 8.75 mm, as well as $0.03\;{\mathrm{mm}}^{-1}$ and 8.75 mm, respectively. Nonetheless, the performances of MSSIM and MISSIM-S demonstrated inconsistency with the contrast ratio and inclusion size. In contrast, MS-ISSIM-S offered stability in measuring the DOT-reconstructed images by performing the reducing of similarity scores following the raising of inclusion contrast, as shown in Figure 13, Figure 14, Figure 15 and Figure 16.

To confirm the performance of these four types of SSIM, the MOS and Spearman’s rank correlation were used, as described in Section 2.5. There were 20 test subjects, none of whom had eye issues, such as color blindness. To measure the MOS, the subjects were shown the reconstructed images, as in Table 2, and provided their opinion in scores in the range of 1 (the inclusion is not detectable) until 5 (the inclusion is very detectable). The experiments were performed under an adjusted illumination and display. These MOS scores were subjective according to the participant’s own individual opinions; thus, they are not credible. However, the MOS scores can imply a relative comparison by using the Spearman’s rank correlation when the comparisons were performed between the similarity scores in four SSIM types with MOS scores.

Table 3 shows the correlation scores for the four SSIM types. MISSIM-S was not appropriate in terms of human visual perception in the case of assessing the DOT-reconstructed images because this technique is designed to overcome the MSSIM underestimation related to translation and scaling and overestimation associated with blurred images. Therefore, MISSIM-S is superior when the comparison is regarding translation and scaling images. However, MS-SSIM is better than MSSIM because it measures images at various scales. Apparently, MS-SSIM works satisfactorily because it can evaluate distorted images well; thus, MS-ISSIM-S was developed in this research based on the advantages of MISSIM-S and MS-SSIM. As shown in Table 3, MS-ISSIM-S presents the highest correlation average score compared to MOS, followed by MS-SSIM, MSSIM, and MISSIM-S. MS-ISSIM-S was more stable in assessing the DOT-reconstructed images with correlation scores from 0.8552 to 0.9955, although the reconstructed images had several image distortions due to the limitations of the algorithm, such as resolution and sensitivity. In addition, MS-SSIM was shown to be promising for the objective assessment of the DOT-reconstructed images, but it had uncertainty in case B1 with 10% noise amplitude, phase, and optical properties, with a correlation score of 0.6864. Nonetheless, the correlation scores were between 0.6864 and 0.9964. MSSIM and MISSIM-S presented unsatisfactory correlations, as it may be observed that MSSIM correlations were 0.6532 to 0.9740 and the MISSIM-S correlations were −0.0974 to 0.9487.

**Figure 17:**
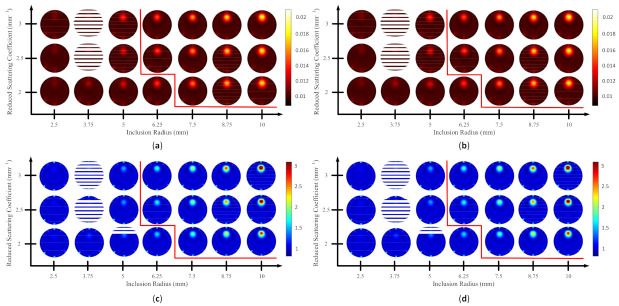
Reconstructed images of case B1 with 1% noise amplitude; red line indicates the invisible (**left side**) and visible (**right side**) inclusions: (**a**) $\mu_{a}$ MS-ISSIM-S, (**b**) $\mu_{a}$ MOS, (**c**) $\mu_{s}’$ MS-ISSIM-S, and (**d**) $\mu_{s}’$ MOS.

**Figure 18:**
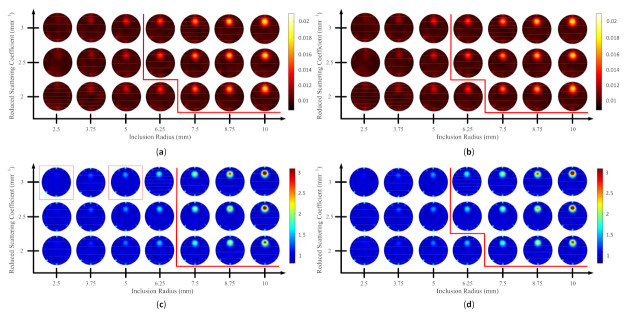
Reconstructed images of case B1 with 10% noise amplitude, phase, and optical properties; red line indicates the invisible (**left side**) and visible (**right side**) inclusions: (**a**) $\mu_{a}$ MS-ISSIM-S, (**b**) $\mu_{a}$ MOS, (**c**) $\mu_{s}’$ MS-ISSIM-S, and (**d**) $\mu_{s}’$ MOS.

**Figure 19:**
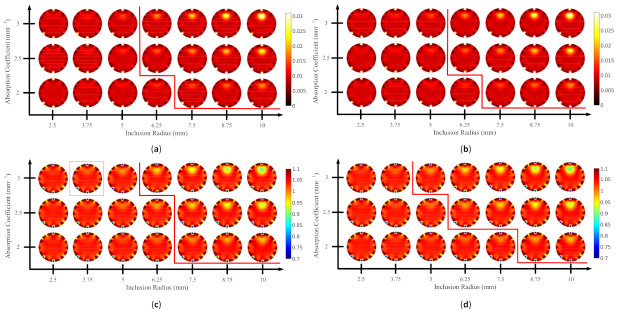
Reconstructed images of case B2 with 1% noise amplitude; red line indicates the invisible (**left side**) and visible (**right side**) inclusions: (**a**) $\mu_{a}$ MS-ISSIM-S, (**b**) $\mu_{a}$ MOS, (**c**) $\mu_{s}’$ MS-ISSIM-S, and (**d**) $\mu_{s}’$ MOS.

**Figure 20:**
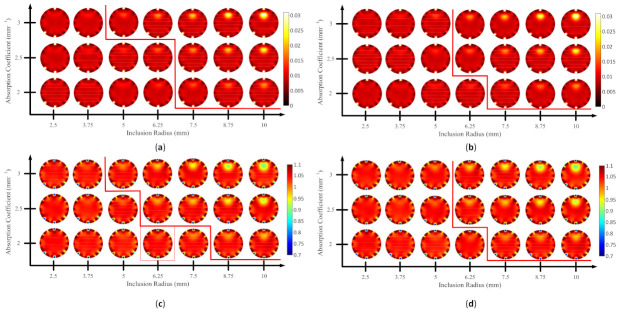
Reconstructed images of case B2 with 10% noise amplitude, phase, and optical properties; red line indicates the invisible (**left side**) and visible (**right side**) inclusions: (**a**) $\mu_{a}$ MS-ISSIM-S, (**b**) $\mu_{a}$ MOS, (**c**) $\mu_{s}’$ MS-ISSIM-S, and (**d**) $\mu_{s}’$ MOS.

Using Spearman’s rank correlation, it is evident that MS-ISSIM-S performed a robust assessment by showing the best correlation scores and suitable values for the contrast and inclusion size, as shown in Table 3 and Figure 13, Figure 14, Figure 15 and Figure 16. Therefore, a median was used as the threshold value to separate the visible and invisible inclusions. Because the ultimate goal of this research was to evaluate the image numerically based on a computer decision, a comparison was performed between MS-ISSIM-S and MOS to validate the algorithm, as shown in Figure 17, Figure 18, Figure 19 and Figure 20.

**Figure 21:**
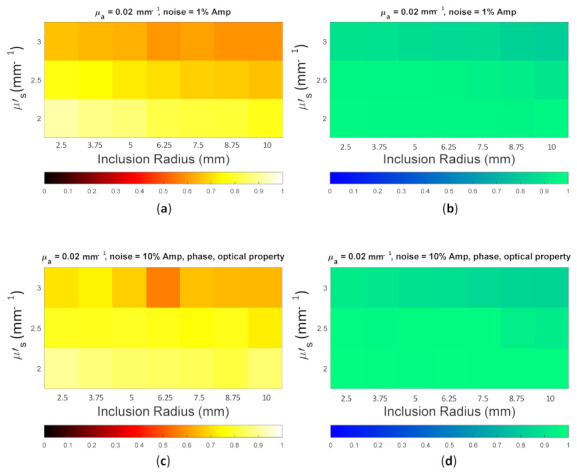
Color map of the MS-ISSIM-S score for (**a**) Figure 17a; (**b**) Figure 17c; (**c**) Figure 18a; and (**d**) Figure 18c.

**Figure 22:**
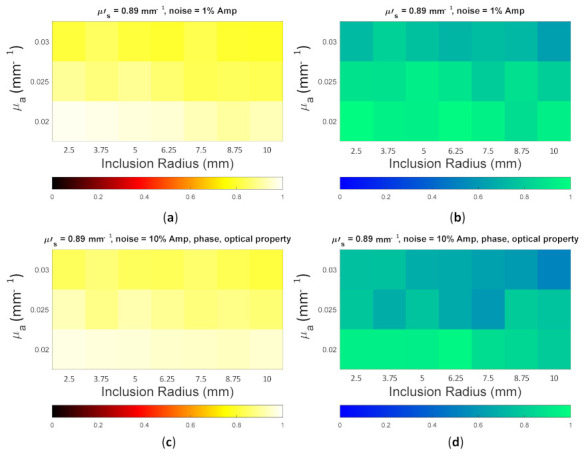
Color map of the MS-ISSIM-S score for (**a**) Figure 19a; (**b**) Figure 19c; (**c**) Figure 20a; and (**d**) Figure 20c.

Figure 17a–d depicts the comparison for case B1 with 1% noise amplitude, as well as the red line to distinguish visible inclusions on the right and invisible on the left. Figure 17 shows that MS-ISSIM-S (Figure 17a,c) had the same results as MOS (Figure 17b,d); thus, in this case, MS-ISSIM-S performed perfectly. Moreover, Figure 18a,b has a similar condition for splitting the detectable and undetectable inclusions. However, for $\mu_{s}^{’},$ case B1 with 10% noise amplitude, phase, and optical properties, as shown in Figure 18c, differed slightly from Figure 18d. Unfortunately, due to aiming to mimic the real environment with adequate noise, Figure 18c shows two errors in assessing the images indicated by the red rectangles. Two red rectangles mean detectable inclusion, whereas the true condition was undetectable inclusion. Yet, to determine a value as a threshold value is not trivial [10]; thus, to determine the algorithm performance, more simulated cases are needed. However, MS-ISSIM-S exhibited more stable results in image assessment of DOT than the other models compared, according to the results, as shown in Table 3. Figure 19a,b has the same results, indicating that MS-ISSIM-S fit with the MOS, whereas Figure 19c depicts one detectable error for size 3.75 mm with $\mu_{s}^{’}=0.89\;{\mathrm{mm}}^{-1}$ and $\mu_{a}=0.03\;{\mathrm{mm}}^{-1}$ compared with Figure 19d. Figure 20a,b has slightly different results because this case had excessive implemented noise. Furthermore, Figure 20c has one detectable error compared to Figure 20d. As shown in Figure 17, Figure 18, Figure 19 and Figure 20, MS-ISSIM-S can be promising in assessing a group of DOT-reconstructed images with low noise. To complete the results, the MS-ISSIM-S similarity scores represented in the color map were recorded. Figure 21 and Figure 22 depict these color maps. Figure 21a–d represents the reconstructed image, as shown in Figure 17a,c and Figure 18a,c, whereas Figure 22a–d demonstrates the reconstructed image, as shown in Figure 19a,c and Figure 20a,c. As can be seen, with lower optical property contrast and small inclusion, such as $\mu_{a}=0.02\;{\mathrm{mm}}^{-1}$, $\mu_{s}^{’}=2\;{\mathrm{mm}}^{-1}$, and inclusion size 2.5 mm, the color map was bright, indicating a high similarity score, and, thus, there was no inclusion. In contrast, with the high contrast ratio and larger inclusion, for instance, $\mu_{a}=0.02\;{\mathrm{mm}}^{-1}$, $\mu_{s}^{’}=3\;{\mathrm{mm}}^{-1}$, and an inclusion size of 10 mm, the similarity score was lower, represented by the dark color, indicating the presence of an inclusion. With these results, SSIM, especially MS-ISSIM-S, shows promise as an option to assess numerically and objectively the image based on computer, regardless of insight in the medical image analysis field. However, experts are necessary to reach a decision in medical applications. Moreover, since we only presented the DOT-reconstructed images from simulation cases, to confirm the results in this paper, clinical image analyses by medical doctors specialized in radiology are necessary to obtain the comprehensive insight of the improved SSIM, especially for MS-ISSIM-S. Once again, the goal of a computer-based observer for medical images is to assist radiologists to achieve a conclusion in the medical field. The comparisons between radiologists’ points of view with the results of this research shortly are essential. In addition, the threshold value here was presented by employing the median for simplicity; hence, further research must consider an appropriate method to conclude this threshold.

## 4. Conclusions

A reconstruction algorithm was implemented to produce DOT-reconstructed images. Simulated cases generating reconstructed images with 1% noise amplitude and 10% noise amplitude, phase, and optical properties were employed. To numerically assess the images, four types of SSIM were used to obtain the similarity scores. To confirm the results, Spearman’s rank correlation was utilized to compare the four SSIMs with MOS. MS-ISSIM-S showed the best correlation, with a score between 0.8552 and 0.9955 and an average correlation of 0.9452, representing a robust image assessment regardless of the noise. A comparison of MOS and MS-ISSIM-S to yield a suitable HVS was performed by separating the image into two sections with the assistance of a threshold value, as indicated graphically by a red line. MS-ISSIM-S demonstrated acceptable results when it measured images with low noise, but the association with HVS was relatively reliable. In addition, with lower optical property contrast and small inclusion, the color map was bright, indicating a high similarity score; thus, there was no inclusion. In contrast, the similarity score of regions with a high contrast ratio and larger inclusion was lower, represented by the dark color; hence, an inclusion was present. These results indicated that SSIM, particularly MS-ISSIM-S, is a promising option for the numerical and objective computational assessment of reconstructed images, regardless of specialized insight in the field of medical image analysis. However, experts naturally remain necessary to make specific medical decisions.

## Figures and Tables

**Table 1:** Simulation cases with the background $\mu_{a}=0.01\;{\mathrm{mm}}^{-1}$ and $\mu_{s}\prime =1\;{\mathrm{mm}}^{-1}$.

Case	Inclusion Radius (mm)	$\mu_{a}\;\left(mm^{-1}\right)$	$\mu_{s}^{’}\left(mm^{-1}\right)$	**Represent**
A1	0	0	0	Homogeneous
	2.5	0.02	2	Invisible
	10	0.02	3	Visible
A2	0	0	0	Homogeneous
	2.5	0.02	0.89	Invisible
	10	0.03	0.89	Visible

**Table 2:** Simulation cases for image assessment.

Case	$\mathrm{Inclusion}\;\mathrm{Radius}\;\left(mm\right)$	$\mu_{a}\;\left(mm^{-1}\right)$	$\mu_{s}^{’}\;\left(mm^{-1}\right)$
B1	2.5/3.75/5/6.25/7.5/8.75/10	0.02	2/2.5/3
B2		0.02/0.025/0.03	0.89

**Table 3:** Correlation by providing the average score using Spearman’s rank between MOS and four types of SSIM.

Case	MSSIM	MISSIM-S	MS-SSIM	MS-ISSIM-S
B1 with 1% noise amplitude	0.9273	0.7636	0.9964	0.9955
	0.9403	0.8545	0.9792	0.9565
B1 with 10% noise amplitude, phase, and optical properties	0.8091	–0.0974	0.6864	0.9623
	0.8630	0.7578	0.9325	0.8552
B2 with 1% noise amplitude	0.9640	0.9487	0.9909	0.9857
	0.9740	0.4146	0.9205	0.9425
B2 with 10% noise amplitude, phase, and optical properties	0.6532	0.2019	0.9792	0.9805
	0.9208	0.5779	0.9481	0.8831
Average	0.8815	0.5527	0.9291	0.9452
